# A high throughput, functional screen of human Body Mass Index GWAS loci using tissue-specific RNAi *Drosophila melanogaster* crosses

**DOI:** 10.1371/journal.pgen.1007222

**Published:** 2018-04-02

**Authors:** Thomas J. Baranski, Aldi T. Kraja, Jill L. Fink, Mary Feitosa, Petra A. Lenzini, Ingrid B. Borecki, Ching-Ti Liu, L. Adrienne Cupples, Kari E. North, Michael A. Province

**Affiliations:** 1 Department of Internal Medicine, Division of Endocrinology, Metabolism and Lipid Research, Washington University School of Medicine, St. Louis, Missouri, United States of America; 2 Department of Genetics and Center for Genome Sciences and Systems Biology, Division of Statistical Genomics, Washington University School of Medicine, St. Louis, Missouri, United States of America; 3 Department of Biostatistics, University of Washington, Seattle, Washington, United States of America; 4 Department of Biostatistics, Boston University School of Public Health, Boston, Massachusetts, United States of America; 5 Department of Epidemiology, University of North Carolina, Chapel Hill, North Carolina, United States of America; Geisel School of Medicine at Dartmouth, UNITED STATES

## Abstract

Human GWAS of obesity have been successful in identifying loci associated with adiposity, but for the most part, these are non-coding SNPs whose function, or even whose gene of action, is unknown. To help identify the genes on which these human BMI loci may be operating, we conducted a high throughput screen in *Drosophila melanogaster*. Starting with 78 BMI loci from two recently published GWAS meta-analyses, we identified fly orthologs of all nearby genes (± 250KB). We crossed RNAi knockdown lines of each gene with flies containing tissue-specific drivers to knock down (KD) the expression of the genes only in the brain and the fat body. We then raised the flies on a control diet and compared the amount of fat/triglyceride in the tissue-specific KD group compared to the driver-only control flies. 16 of the 78 BMI GWAS loci could not be screened with this approach, as no gene in the 500-kb region had a fly ortholog. Of the remaining 62 GWAS loci testable in the fly, we found a significant fat phenotype in the KD flies for at least one gene for 26 loci (42%) even after correcting for multiple comparisons. By contrast, the rate of significant fat phenotypes in RNAi KD found in a recent genome-wide Drosophila screen (Pospisilik et al. (2010) is ~5%. More interestingly, for 10 of the 26 positive regions, we found that the nearest gene was not the one that showed a significant phenotype in the fly. Specifically, our screen suggests that for the 10 human BMI SNPs rs11057405, rs205262, rs9925964, rs9914578, rs2287019, rs11688816, rs13107325, rs7164727, rs17724992, and rs299412, the functional genes may NOT be the nearest ones (*CLIP1*, *C6orf106*, *KAT8*, *SMG6*, *QPCTL*, *EHBP1*, *SLC39A8*, *ADPGK /ADPGK-AS1*, *PGPEP1*, *KCTD15*, *respectively*), but instead, the specific nearby cis genes are the functional target (namely: *ZCCHC8*, *VPS33A*, *RSRC2; SPDEF*, *NUDT3; PAGR1; SETD1*, *VKORC1; SGSM2*, *SRR; VASP*, *SIX5; OTX1; BANK1; ARIH1; ELL; CHST8*, respectively). The study also suggests further functional experiments to elucidate mechanism of action for genes evolutionarily conserved for fat storage.

## Author summary

Human Genome Wide Association Studies have successfully found thousands of novel genetic variants associated with many diseases. While these undoubtedly point to new biology, the field has been slowed in exploiting these new findings to reach a better understanding of exactly how they confer increased risk. Many, if not most, appear to be regulatory not coding variants, so their immediate consequence is not obvious. A real rate limiting step is even identifying which gene these variants might be regulating, and in what tissues they are operating to increase disease risk. In the absence of any other information, a first order assumption is that they may be more likely to be regulating a nearby gene, and such variants are often initially annotated by the “nearest” gene until their function is more definitively validated. Exploiting the idea that many genes may have conserved function across species, we conducted a high-throughput screen of fruit-fly orthologs of human genes nearby 78 well validated GWAS variants for human obesity, in order to more precisely identify the gene(s) of action. We systematically knocked down the function of each of these nearby genes in the brain and fat-body of the flies, raised them on a standard diet, and compared their percent body fat with control flies, in order to validate which genes showed a fat response. 43% of the time when fly orthologs existed in the region, we were able to identify the causal gene. Interestingly, nearly half the time (46%), it was not the nearest gene but another nearby one that regulated fat.

## Introduction

Human Genome-Wide Association Scans (GWAS) have been successful in discovering many genetic loci that are significantly associated with Body Mass Index (BMI). These associations have been replicated across consortia consisting of many large and independent studies, sometimes numbering into hundreds of thousands of subjects. From a statistical point of view, the evidence that single nucleotide polymorphisms (SNPs) are tagging something real is overwhelming. However, progress has been slowed in moving beyond the discovery phase to a deeper understanding the biological significance of these findings, due to difficulties isolating the driving causal variants or even identifying the acting genes tagged by these GWAS variants. Most of the findings are not in gene coding regions. Indeed, many are intergenic, suggesting that much of the underlying modes of action of these loci may be regulatory. Unfortunately, our limited biological understanding of the regulome has hampered further progress.

While the field has begun serious annotation of the regulatory regions of the genome with initiatives and resources such as ENCODE, RoadMap and GTEx, the annotation is still far from complete and the answers that are emerging are complex. As a result, many publications annotate the statistically significant SNPs simply with the “closest” gene, even though trans-acting regulatory sequences certainly exist[1], and some enhancers have been shown to regulate multiple genes[2]. Recently, it was reported that rs1421085 T-to-C intron of the well-known obesity-associated *FTO* gene, disrupts a conserved motif for the *ARID5B* repressor, which leads to derepression of a potent preadipocyte enhancer and a doubling of the transcription factors *IRX3* and *IRX5* expression during early adipocyte differentiation. *Irx3*-deficient mice showed a 25–30% reduction of body weight, primarily through the loss of fat mass and increase in basal metabolic rate. Hypothalamic expression of a dominant-negative form of *Irx3* reproduces the metabolic phenotypes of *Irx3*-deficient mice. Thus, *IRX3* has been suggested as a functional long-range target of obesity-associated variants within *FTO* and represents a novel determinant of body mass and composition, by regulating the process of thermogenesis as they can prevent the process in which energy is turned into heat, thus stored as fat [3, 4].

**Fig 1:**
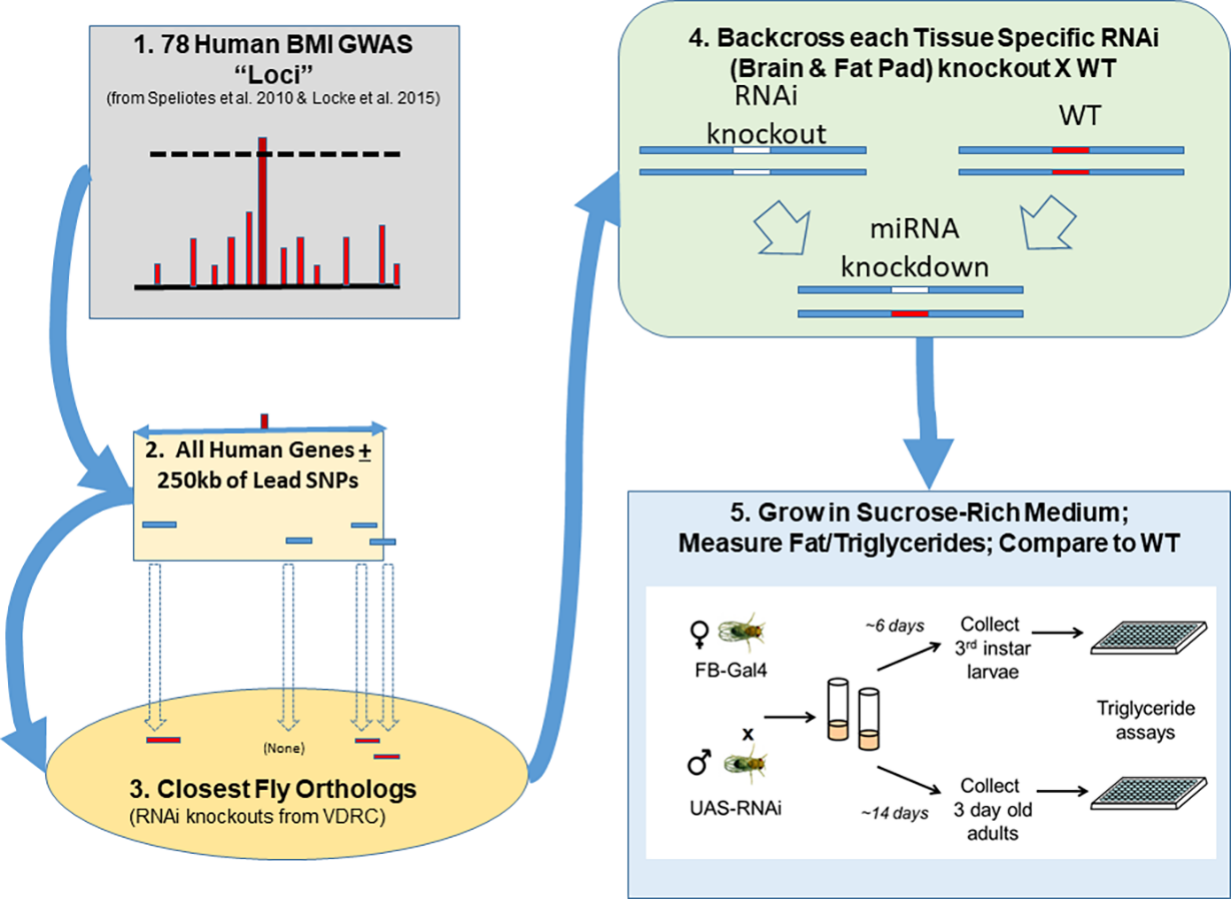
Experimental design of the Drosophila BMI loci functional screen. WT = Corresponding RNAi Wild Type (as detailed in Fig 3).

The above research supports the idea that an intronic location of an associated SNP does not even establish that the genetic effect is on that gene. Carrying out functional mapping of these GWAS-associated regions can provide valuable information to sort out which genes are causal to adiposity, and possibly provide biological insight into their action. While mouse models of obesity can serve as powerful platforms to functionally probe a small number of candidate genes, this approach is expensive and time consuming, limiting the number of genes that can be readily assessed. However, many important biochemical pathways involved in growth, metabolism, fat storage and retrieval are ancient and are therefore well conserved across the animal kingdom from *C*. *elegans* and *Drosophila* to rodents and humans. For example, forward genetic screens in *C*. *elegans* and *Drosophila* have identified conserved genes that regulate triglyceride storage[5, 6]. Readily available genetic tools in *Drosophila*, including mutations and inducible RNA interference (RNAi), coupled with the short life span, offer the opportunity for high-throughput functional screening of candidate genes, such as those proximal to GWAS putatively regulatory variants. A *Drosophila* genetic approach was recently used to follow up a small-scale GWAS for Alzheimer pathology[7] and type 2 diabetes mellitus and related metabolic disorders[8]. Capitalizing on this approach, we conducted a high throughput functional screen in *Drosophila* of all nearby genes to 78 BMI SNPs from two recently published GWAS meta-analyses to see if we could make progress in identifying the possible genes of action for these novel loci, as outlined in Fig 1.

## Results

In Table 1, we show the specific qualitative results of the Drosophila screen for each of the 78 index BMI SNPs (detailed results for each gene are given in S1 Table). For 16 (21%) of the BMI loci, the 500-kb region around the locus could not be interrogated with our Drosophila screen, as none of those genes had a fly ortholog. Details for these unscreened loci and regions are listed in Table 1. For the remaining 62 (79%) loci, at least one gene in the 500-kb region had a fly ortholog which could be KD in our screen. Details of the results for these 62 loci are given in Table 1. In Table 1, we show the lead SNP identified by GWAS, its BMI association *P*-value (as previously reported), the first author of the reference paper identifying that SNP association, its position (per db147), the nearest gene, the annotation of the role of the SNP (if within the nearest gene), and finally we list all other genes within 250 kb radius of the lead SNP. From Table 1, a total of 439 genes were found within the queried intervals of these 78 SNPs. 224 (51%) of those genes had fly orthologs with transgenic RNAi stocks available. Additionally, 30 (13%) of the RNAi KD crosses were lethal (or pupal lethal), and thus could not be evaluated for %BF phenotype. Overall, 36 RNAi gene KDs showed significantly higher or lower %BF at adulthood compared to controls, using Dunnett’s Multiple Comparison test, which accounts for multiple comparisons with common controls.

**Table 1:** Results of the functional drosophila screen for all genes nearby (± 250 kb) the 78 BMI-associated GWAS loci from speliotes et al. 2010[30] and locke et al. 2015[31].

Published Human BMI GWAS Loci						Nearest Gene to SNP (GRCh37.p13)		Additional Nearby Genes (within + 250 kb of SNP)		
#	SNP	BMI p-value	Pub	Chr	SNP bp position (db147)	Gene Name	SNP Role (if in gene)	Tested Significant %BF in Fly	Tested Not Significant %BF in Fly	Could Not be Tested in Fly*
***Nearest Gene Significant for Fly %BF Differences from Control (N = 11 Loci)***										
1	rs977747	2.18E-08	L	1	47219005	*TAL1* ^a^	utr-3’	*CYP4A22* ^a^, *FOXD2/ FOXD2-AS1* ^a^	*CYP4X1*, *CMPK1*	*CYP4Z1*, *LINC00853*, *PDZK1IP1*, *FOXE3*, *STIL*
2	rs7903146	1.11E-11	L	10	112998590	*TCF7L2* ^a^	intron	*VTI1A* ^a^		
3	rs11583200	1.48E-08	L	1	50094148	*ELAVL*4 ^a^	intron			
4	rs7599312	1.17E-10	L	2	212548507	*ERBB4* ^a^				
5	rs9400239	1.61E-08	L	6	108656460	*FOXO3* ^a^	utr-5’		*LACE1*, *ARMC2*	*LINC00222*
6	rs13191362	7.34E-09	L	6	162612318	*PARK2* ^a^	intron		*PACRG*	
7	rs2033732	4.89E-08	L	8	84167474	*RALYL* ^a^				
8	rs11191560	8.45E-09	L	10	103109281	*NT5C2* ^a^	intron		*C10orf32*, *CNNM2*	*AS3MT*, *INA*, *PCGF6*
9	rs2176598	2.97E-08	L	11	43842728	*HSD17B12* ^a^	intron			*ACCS*, *ACCSL*, *ALKBH3*, *C11orf96*
10	rs10150332	2.75E-11	S	14	79470621	*NRXN3* ^a^	intron			
11	rs2650492	1.92E-09	L	16	28322090	*SBK1* ^a^	utr-3’		*XPO6*, *CCDC101*, *CLN3*, *NUPR1*	*EIF3CL*^b^, *APOBR* *IL27*
***Nearest Gene Tested Not Significant for Fly %BF*, *but other Nearby Gene(s) are Significant (N = 10 Loci)***										
12	rs11057405	2.02E-08	L	12	122297350	*CLIP1*	intron	*ZCCHC8* ^a^, *VPS33A* ^a^, *RSRC2* ^a^	*KNTC1*	*B3GNT4*, *DIABLO*, *IL31*, *LRRC43*
13	rs205262	1.75E-10	L	6	34595387	*C6orf106*	intron	*SPDEF* ^a^, *NUDT3* ^a^	*PACSIN1*, *SNRPC*, *UHRF1BP1*	*RPS10*^b^, *RPS10-NUDT3*
14	rs9925964	8.11E-10	L	16	31118574	*KAT8*	intron	*SETD1A* ^a^, *VKORC1* ^a^	*BCL7C*, *HSD3B7*, *FUS*	*ORAI3*^b^, *STX1B*^b^, *BCKDK*, *CTF1*, *FBXL19/FBXL19-AS1*, *MIR4519*, *PRSS53*, *STX4*, *ZNF646*, *ZNF668*, *C16orf98*, *ITGAM*, *ITGAX*, *PRSS36*, *PRSS8*, *PYCARD*, *PYDC1*, *TRIM72*
15	rs9914578	2.07E-08	L	17	2101842	*SMG6*	intron	*SGSM2* ^a^, *SRR* ^a^	*DPH1*	*RPA1*^b^, *TSR1*^b^, *HIC1*, *MIR132*, *MIR212*, *RTN4RL1*, *SNORD91A*, *SNORD91B*
16	rs2287019	1.88E-16	S	19	45698914	*QPCTL*		*VASP* ^a^, *SIX5* ^a^	*EML2*, *RTN2*, *DMPK*, *DMWD*, *IRF2BP1*, *NANOS2*, *RSPH6A*	*ERCC1*^b^, *FOSB*^b^, *OPA3*^b^, *SNRPD2*^b^, *NOVA2*^b^, *SYMPK*^b^, *GIPR*, *GPR4*, *PPM1N*, *FBXO46*, *FOXA3*, *LOC388553*, *MYPOP*
17	rs11688816	1.89E-08	L	2	62825913	*EHBP1*	intron	*OTX1* ^a^		
18	rs13107325	1.50E-13	S	4	102267552	*SLC39A8*	mis-sense	*BANK1* ^a^	*NFKB1*	
19	rs7164727	3.92E-09	L	15	72801650	*ADPGK/ ADPGK-AS1*		*ARIH1* ^a^	*BBS4*, *HIGD2B*	*GOLGA6B*, *LOC646670*, *MIR630*
20	rs17724992	3.42E-08	L	19	18344015	*PGPEP1*	intron	*ELL* ^a^	*IFI30*, *JUND*, *LSM4*, *MAST3*, *MPV17L2*, *PDE4C*, *PIK3R2*, *RAB3A*, *FKBP8*, *ISYNA1*, *KXD1*, *SSBP4*	*UBA52*^b^, *KIAA1683*, *LOC729966*, *C19orf60*, *CRLF1*, *GDF15*, *LRRC25*
21	rs29941	3.01E-09	S	19	33818627	*KCTD15*		*CHST8* ^a^		
Nearest Gene Could Not be Tested in Fly*, *but other Nearby Gene(s) are Significant (N = 5 Loci)*										
22	rs4787491	2.70E-08	L	16	30004016	*INO80E*	intron	*YPEL3* ^a^, *PAGR1* ^a^	*ASPHD1*, *CDIPT*, *KCTD13*, *TAOK2*, *CORO1A*, *DOC2A*, *FAM57B*, *MAPK3*, *TBX6*	*ALDOA*^b^, *BOLA2B*^b^, *PPP4C*^b^, *HIRIP3*, *KIF22*, *LOC100289283*, *MAZ*, *MVP*, *PRRT2*, *SEZ6L2*, *TMEM219*, *ZG16*, *C16orf92*, *GDPD3*, *SLX1A*, *LOC613037*, *LOC613038*, *SLX1A-SULT1A3*
23	rs7138803	1.82E-17	S	12	49853685	*BCDIN3D/ BCDIN3D-AS1*		*RACGAP1* ^a^	*FMNL3*, *PRPF40B*, *TMBIM6*, *AQP2*, *AQP5*,	*SMARCD1*^b^, *FAM186B*, *NCKAP5L*, *AQP6*, *ASIC1*, *FAIM2*
24	rs6465468	4.98E-08	L	7	95540202	*ASB4*	utr-3’	*PDK4* ^a^	*PPP1R9A*	*DYNC1I1*, *PON1*, *PON2*, *PON3*
25	rs16907751	3.89E-08	L	8	80463222	*ZBTB10*		*ZNF704* ^a^		*MIR5708*
26	rs758747	7.47E-10	L	16	3577357	*NLRC3*	utr-5’	*CLUAP1* ^a^	*NAA60*	*C16orf90*, *MTRNR2L4*, *OR2C1*, *ZNF174*, *ZSCAN32*, *DNASE1*, *SLX4*
***Nearest Gene Tested Not Significant for Fly %BF and No Significant Fly %BF Gene in Region (N = 22 Loci)***										
27	rs657452	5.48E-13	L	1	49124175	*AGBL4*	intron			
28	rs2815752	1.61E-22	S	1	72346757	*NEGR1*				
29	rs12401738	1.15E-10	L	1	77981077	*FUBP1*	intron		*DNAJB4*, *FAM73A*, *USP33*, *GIPC2*	*NEXN /NEXN-AS1*, *MGC27382*
30	rs543874	3.56E-23	S	1	177920345	*SEC16B*				*LOC730102*, *RASAL2 /RASAL2-AS1*
31	rs2820292	1.83E-10	L	1	201815159	*NAV1*	intron		*IPO9*, *LMOD1*, *TIMM17A*	*MIR1231*, *MIR5191*, *ELF3*, *RNPEP*, *SHISA4*
32	rs2867125	2.77E-49	S	2	622827	*TMEM18*				*LOC100996637*, *LOC727944*
33	rs11126666	1.33E-09	L	2	26705943	*KCNK3*	intron		*DRC1*, *OTOF*, *DPYSL5*, *SLC35F6*	*C2orf70*, *CIB4*, *CENPA*
34	rs2890652	1.35E-10	S	2	142202362	*LRP1B*				
35	rs2365389	1.63E-10	L	3	61250788	*FHIT*	intron			
36	rs3849570	2.60E-08	L	3	81742961	*GBE1*	intron			
37	rs16851483	3.55E-10	L	3	141556594	*RASA2*	intron		*RNF7*	*ZBTB38*, *GRK7*, *LOC646730*
38	rs17001654	7.76E-09	L	4	76208415	*SCARB2*	intron		*NUP54*, *SHROOM3*	*SDAD1*^b^, *ART3*, *CXCL11*, *CXCL9*, *CCDC158*, *FAM47E*, *FAM47E-STBD1*
39	rs1167827	6.33E-10	L	7	75533848	*HIP1*	utr-3’			*LOC541473*, *NSUN5P1*, *PMS2L2*, *PMS2P3*, *POM121C*, *SPDYE5*, *STAG3L1*, *TRIM73*, *CCL26*
40	rs9641123	2.08E-10	L	7	93568420	*CALCR*	intron		*CCDC132*,	*MIR4652*
41	rs10733682	1.83E-08	L	9	126698635	*LMX1B*	utr-3’		*MVB12B*, *RALGPS1*	*ZBTB34*, *ZBTB43*
42	rs3817334	1.59E-12	S	11	47629441	*MTCH2*	intron		*CELF1*, *NDUFS3*, *PSMC3*, *PTPMT1*, *RAPSN*, *SLC39A13*, *AGBL2*	*NUP160*^b^, *C1QTNF4*, *FAM180B*, *KBTBD4*, *MIR4487*, *FNBP4*
43	rs1441264	2.96E-08	L	13	79006784	*RBM26*				
44	rs3736485	7.41E-09	L	15	51456413	*DMXL2*	intron		*GLDN*	*CYP19A1*, *SCG3*
45	rs12444979	2.91E-21	S	16	19922278	*GPRC5B*			*C16orf62*	*IQCK*, *KNOP1*
46	rs1000940	1.28E-08	L	17	5379957	*RABEP1*	intron		*USP6*, *C1QBP*, *DERL2*, *RPAIN*	*DHX33*^b^, *NUP88*^b^, *SCIMP*, *ZNF594*, *LOC728392*, *MIS12*, *NLRP1*
47	rs3810291	1.64E-12	S	19	47065746	*ZC3H4*	utr-3’		*AP2S1*, *ARHGAP35*, *NPAS1*, *SAE1*	*SNAR-E*, *TMEM160*, *BBC3*, *C5AR1*, *CCDC9*, *PRR24*
48	rs1808579	4.17E-08	L	18	23524924	*NPC1 overlapping* *C18orf8*	intron		*RIOK3*, *LAMA3*, *ANKRD29*	*TMEM241*,
Nearest Gene Could Not be Tested in Fly* ***and No Significant Fly %BF Gene in Region (N = 14 Loci)***										
49	rs3888190	3.14E-23	L	16	28878165	*ATP2A1*^b^			*ATXN2L*, *SH2B1*, *SPNS1*	*TUFM*^b^, *EIF3C*, *MIR4721*, *CD19*, *LAT*, *MIR4517*, *NFATC2IP*, *RABEP2*, *RRN3P2*
50	rs2075650	1.25E-08	L	19	44892362	*TOMM40*^b^	intron		*CBLC*, *RELB*, *ZNF296*,	*CLPTM1*^b^, *PPP1R37*^b^, *BCAM*, *BCL3*, *CEACAM16*, *CEACAM19*, *MIR4531*, *PVR*, *PVRL2*, *APOC1*, *APOC1P1*, *APOC2*, *APOC4*, *APOE*, *CLASRP*, *GEMIN7*
51	rs17203016	3.41E-08	L	2	207390794	*MIR1302-4*			*KLF7*, *CREB1*	*METTL21A*
52	rs492400	6.78E-09	L	2	218485029	*USP37*	intron		*ARPC2*, *CTDSP1*, *SLC11A1*, *VIL1*, *BCS1L*, *RQCD1*, *STK36*, *TTLL4*	*AAMP*^b^, *RNF25*^b^, *GPBAR1*, *PNKD*, *PLCD4*, *ZNF142*
53	rs9816226	1.69E-18	S	3	186116710	*ETV5*			*TRA2B*, *DGKG*	*LOC344887*
54	rs2112347	2.17E-13	S	5	75719417	*POC5*			*COL4A3BP*	*ANKDD1B*, *POLK*
55	rs4740619	4.56E-09	L	9	15634328	*CCDC171*	intron		*PSIP1*, *SNAPC3*	
56	rs17094222	5.94E-11	L	10	100635683	*HIF1AN*			*NDUFB8*, *PAX2*	*SEC31B*^b^, *LINC00263*, *WNT8B*
57	rs4256980	2.90E-11	L	11	8652392	*TRIM66*	intron			*RPL27A*^b^, *STK33*, *ST5*
58	rs10767664	4.69E-26	S	11	27704439	*BDNF/* *BDNF-AS1*	intron		*LGR4*, *LIN7C*	
59	rs2241423	1.19E-18	S	15	67794500	*MAP2K5*	intron		*SKOR1*	*RNU6-1*
60	rs11074446	1.71E-10	L	16	20243801	*UMOD*			*PDILT*	*GPR139*, *ACSM2A*, *ACSM5*
61	rs2836754	1.61E-08	L	21	38919816	*FLJ45139*			*ETS2*	*LINC00114*, *PSMG1*
62	rs7243357	3.86E-08	L	18	59216087	*GRP*			*LMAN1*, *RAX*	*SEC11C*^b^, *ZNF532*, *CCBE1*, *CPLX4*
***No Fly Orthologs to Any Genes in Region (N = 16 Loci)***										
63	rs1514175	8.16E-14	S	1	74525960	*FPGT-TNNI3K*	intron			*C1orf173*, *CRYZ*, *TNNI3K*, *TYW3*
64	rs887912	1.79E-12	S	2	59075742	*FLJ30838*				
65	rs6804842	2.48E-09	L	3	25064946	*LOC100505947*	intron			*RARB*
66	rs13078807	3.94E-11	S	3	85835000	*CADM2*	intron			
67	rs11727676	2.55E-08	L	4	144737912	*HHIP /* *HHIP-AS1*	intron			
68	rs2033529	1.39E-08	L	6	40380914	*TDRG1*				*LRFN2*
69	rs13201877	4.29E-08	L	6	137354404	*IFNGR1*				*IL22RA2*, *OLIG3*
70	rs10968576	2.65E-13	S	9	28414341	*LINGO2*	intron			
71	rs1928295	7.91E-10	L	9	117616205	*TLR4*				*ASTN2*
72	rs7899106	2.96E-08	L	10	85651147	*GRID1 /GRID1-AS1*	intron			
73	rs12286929	1.31E-12	L	11	115151684	*CADM1*				
74	rs12429545	1.09E-12	L	13	53528071	*LINC00558*				
75	rs10132280	1.17E-10	L	14	25458973	*STXBP6*				
76	rs2080454	8.60E-09	L	16	49028679	*CBLN1*				
77	rs1558902	4.80E-120	S	16	53769662	*FTO*	intron			*RPGRIP1L*
78	rs571312	6.43E-42	S	18	60172536	*MC4R*				

Legend

# = BMI Locus Number

Pub = Publication identifying the BMI Locus (L = Locke et al., 2015; S = Speliotes et al., 2010)

%BF = Percent Body Fat

*Could Not be Tested in Fly = either No Fly Ortholog to Human Gene, No RNAi KO Fly line available, or RNAi KD of Fly Ortholog Fatal

^*a*^ = RNAi KD of Fly tested significant %Body Fat compared to Control by Dunnett’s Multiple Comparison Test

^*b*^ = RNAi KD of Fly Ortholog Gene FATAL (could not be functionally screened in Fly)

**Fig 2:**
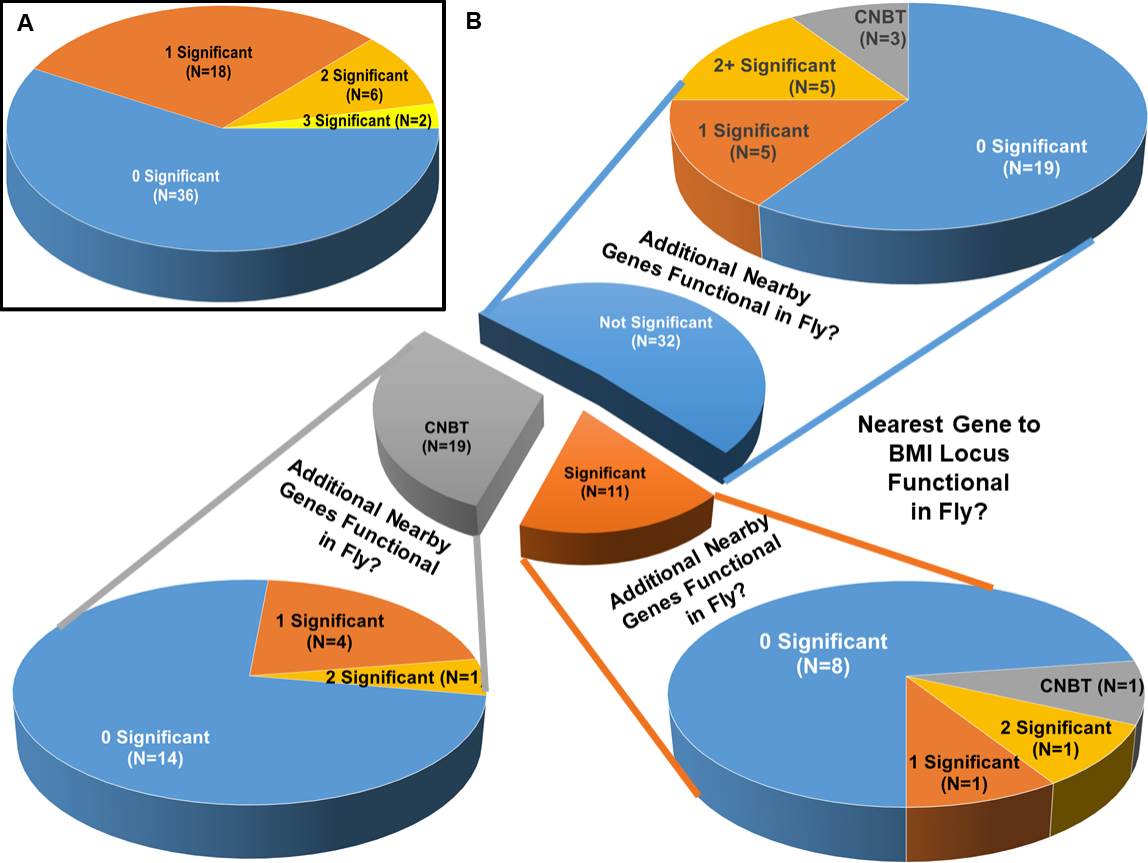
Summary of Drosophila functional scan for the 61 BMI GWAS loci that could be tested in the fly. Number of GWAS BMI loci for which nearby genes were validated in RNAi KDs in Drosophila. **(a)** Distribution of Number of Significant Fly KD Genes per BMI Locus Region. Significance determined by Dunnett’s Multiple Comparisons Test. **(b)** Number of Significant Fly KD Genes per BMI Locus by Proximity to SNP. Significance determined by Dunnett’s Multiple Comparisons Test. CNBT = Could Not Be Tested in Fly (either No Fly Ortholog or KD lethal).

The results for the screened loci from Table 1 are summarized in Fig 2. In Fig 2A, we show that 26 of the 62 screened loci (42%), have at least one significant KD phenotype gene in the nearby region (± 250 kb). For 18 of these loci, there was only one gene significant, whereas we found 2 significant genes producing phenotypes in 6 regions and 3 significant genes producing phenotype in 2 of the regions (for a total of 36 significant genes).

Since the nearest gene is commonly used to annotate a significantly associated SNP, we show the yield from our screen for proximal vs. more distal genes in Fig 2B. Considering only the nearest genes: 45 (74% of the original 78 loci) had fly orthologs that could be phenotyped, while 16 (26%) either had no fly ortholog, or the KD was lethal. Of the 45 nearest genes that could be assessed in the fly, 11 (24%), tested as having significantly different %BF than controls by Dunnett’s Multiple Comparison test. For 9 of these 11 significant loci, no other gene tested in the 500kb region showed a significant phenotype, suggesting that the nearest gene may indeed represent the functional target of the original GWAS SNP. These 9 genes are: *ELAVL4*, *ERBB4*, *FOXO3*, *PARK2*, *RALYL*, *NT5C2*, *HSD17B12*, *NRXN3* and SBK1. For 3 of the 11 significant nearest genes, one (*TCF7L2*) had an additional nearby gene (within 250kb radius of the BMI SNP) with a significant phenotype (*VTI1A*); another significant nearest gene (*TAL1*) had two neighboring genes (within 250KB radius of the BMI lead-SNP) with significant phenotypes (*CYP4A22*, *FOXD2/FOXD2-AS1*); and the third significant nearest gene (HSD17B2) had no additional nearby candidates that had a fly ortholog.

Interestingly, for 5 BMI-associated SNPs, the nearest gene was testable in the fly, but did not show a significant difference in %BF from control when knocked down. However exactly one other gene in the 500-kb region of the lead SNP did show significant %BF differences from control. These 5 significant genes are *OTX1*, *BANK1*, *ARIH1*, *ELL*, and *CHST8*. Our experiment would suggest that the corresponding lead SNPs may be tagging one or more regulatory elements functionally operating on these more distal genes and not the most proximal ones. Additionally, there were 5 SNPs where the closest gene could not be screened in the fly (either because there is no ortholog RNAi line available, or the KD is lethal), but one or more genes in the 500-kB region showed a significant %BF change from control. These 6 significant genes from the 5 regions are *YPEL3*, *PAGR1*, *RACGAP1*, *PDK4*, *ZNF704*, *CLUAP1*. These may also be regions where the more distal gene is the target (but the evidence is less clear, since the nearest gene could not be tested in the fly). Finally, there were 8 SNPs for which multiple genes in the 500-kb region showed a significant KD phenotype, suggesting that perhaps the original SNP finding may be reflecting combined signals from multiple obesity genes.

**Fig 3:**
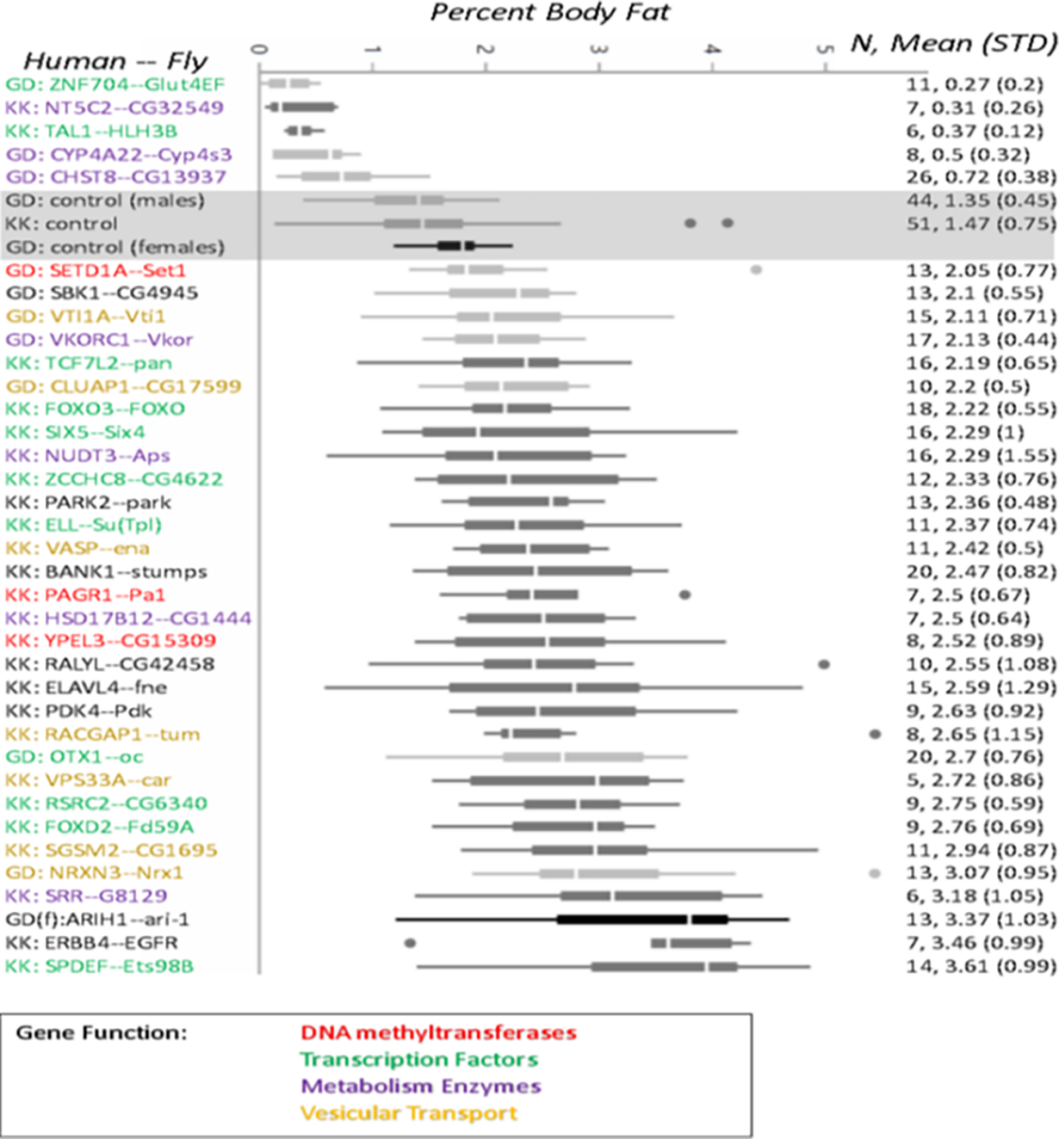
Percent body fat distributions for the 36 RNAi knock down Drosophila gene crosses, testing significantly different from corresponding Wild Type control, by Dunnett’s multiple comparisons test.

Quantitative details of the results for the 36 significant genes are shown in Fig 3. We display the mean and standard deviation of %BF at adulthood for each gene KD, along with box-plots depicting the distributions of the data. For each of the KD gene lines, we give the corresponding human gene, and identify which fly control background (GD or KK) was used to assess significance, under the same feeding and environmental lab conditions. The largest significant changes were found for the *SPDEF* ortholog (Ets98B) KD, which showed an average 2.46-fold increase in %BF, and for the *ZNF704* ortholog (Glut4EF) KD, which showed a 5-fold decrease in %BF. As can be seen, the experiment was able to detect significant increases of at least 1.5 fold and decreases of at least 1.8 fold in specific KD lines.

In Fig 3, we also annotate the major classes of genes demonstrating significant %BF changes. Three of the 36 significant genes are DNA methyltransferases (*SETD1A*, *PAGR1*, and *YPEL3*), 9 are transcription factor genes *(ZNF704*, *TAL1*, *TCF7L2*, *FOXO3*, *SIX5*, *ZCCHC8*, *OTX1*, *FOXD2*, and *SPDEF*), 7 are metabolism enzymes (*NT5C2*, *CYP4A22*, *CHST8*, *VKORC1*, *NUDT3*, *HSD17B12*, and *SRR*), and 7 vesicular transport genes (*VTI1A*, *CLAP1*, *VASP*, *RACGAP1*, *SGSM2* and *NRXN3*), and the remaining 9 have different functions (*RSRC2*, *SBK1*, *PARK2*, *BANK1*, *RALYL*, *ELAVL4*, *PDK4*, *ARIH1*, and *ERBB4*) shown in Fig 3.

### Functional validation in the mouse

To further validate the Drosophila functional results, we queried several bioinformatic resources, including the Mouse Genome Informatics (MGI) website (http://www.informatics.jax.org/), which catalogues publication results of mouse experiments, as well as the International Mouse Phenotyping Consortium (IMPC) website (http://www.mousephenotype.org/), which also contains unpublished as well as published extensive phenotype characterizations screens of knock out (KO) experiments (the results are summarized in S2 Table). At the IMPC website, at this time, there are results of whole organism knockdown experiments for only 8 of our 36 human-fly obesity genes (many more are planned in the future). One of these, SBK1, was pre-weening lethal as a whole organism knockdown, and thus could not be evaluated. For the other seven, there were extensive phenotypic characterizations of adult mice, including Dexa fat mass evaluations. Three of these genes showed highly statistically significant fat mass differences between the KO and WT mice: SETD1A (P = 2.46E-08), TCF7L2 (P = 5.97E-10) and FOXO3 (P = 3.41E-05) while two were nearly statistically significant different from WT: ZNF704 (P = 0.086) and YPEL3 (P = 0.082) and two showed no differences from WT (CLUAP1 and PDK4). Thus, 5 of the 8 of our genes that have thus far been interrogated by whole body KO in mice in the IMPC confirm our results. Obesity-related phenotypes have been previously published for two of the three most significant genes. TCF7L2 KO mice were shown to be leaner and have improved glucose tolerance[9], and further, TCF7L2 was shown to negatively regulate adipocyte differentiation[10]. FOXO3 has been shown to be downregulated in the brains of high-fat diet induced obese mice[11] and mRNA of FOXO3 levels were associated with chicken growth traits, including fat body weight[12]. The MGI website listed confirmatory mouse-obesity evidence for several of the same genes as the IMPC, but no additional ones. In literature search of PubMed, we found suggestive or validation evidence for 5 more of the 36 human-fly obesity genes. Whole body siRNA of NTSC2 in mice showed increased lipolysis [13] and VTI1A was shown to interact with GLUT4 in adipocytes in mice[14]. More directly, PARK2 KO mice show decreased fat absorption and are leaner on high-fat diets[15]. VASP KO mice have reduced body weight and increased brown adipocytes[16]. They also show increased triglyceride accumulation in liver[17]. Finally, mouse expression and fly KDs confirm our findings for NUDT3 by Williams et al. 2015[18]. NUDT3 was significantly up regulated in the reward and feeding related regions of the hypothalamus and amygdala of the mouse brain. In all, we found validation evidence supporting obesity phenotypes in mice for 10 of the 36 genes found in our fly validation screen of cis genes near human BMI loci (ZNF704, SETD1A, VTI1A, TCF7L2, FOXO3, NUDT2, PARK2, VASP, and YPEL3).

However, it should be noted that few of the published or unpublished catalogued experiments, if any, actually reproduce the exact conditions of our fly screen. The IMPC KO screen, as well as most of the published mouse data available are on whole organism knock outs, whereas in our fly screen, we used tissue-specific drivers to confine the knock down only to the brain and to adipose tissue. This is an extremely important difference. Whole organism knock out animals typically experience a wide range of phenotypes across many systems and organs, and in some cases, very severe defects (some are even lethal). Thus, the lack of concordance in obesity phenotypes in such experiments does not mean that our fly experiment has been properly tested for replication in the mouse and it failed. Rather, the fact that we already have found suggestive or strong evidence for 10 of the 36 of the fly genes in the mouse, demonstrates the utility of using the fly as a high-throughput functional screen to help us identify which genes the non-coding human GWAS statistical loci might be regulating, as an important step to moving from statistical association to mechanism of action.

### eQTL validation in human tissues

We used the GTEX resource to see if there is any evidence that the 78 BMI loci are eQTLs for any of the 36 human-fly obesity genes, in the relevant human tissues (S2 Table). We considered 6 human tissues available in GTEX: Adipose Subcutaneous, Adipose Omentum, and Liver (corresponding to the fly fat body), and Brain Hypothalamus, Brain Hippocampus and Pancreas (corresponding to the fly brain—the last because clusters of cells in the brain of flies secrete insulin). We find 19 of the 36 gene-loci pairs show significant eQTL evidence in at least one relevant human tissue.

## Discussion

There is a vast literature on the genetics of fat in Drosophila, using many different experimental approaches [19–21]. In fact, we find 1,198 references to [“Drosophila” AND “fat” AND “genetics”] in Pubmed. Some of this literature shows some of the same genes regulating fat storage as we find in our human-to-fly screen (e.g. *FOXO3*[22]). However, our primary goal here is not to add to knowledge about Drosophila biology per se, but rather to use the fly model as an efficient screen to help the human genetics field move from GWAS statistical “loci” of largely unknown function, to identification of the gene of action, in cases where the current human annotation is unclear or ambiguous.

In a similar experiment to the one we report here, in which the goal was to elucidate GWAS findings for diabetes, Baranski *et al*. studied 38 human genomic regions in which SNPs were associated with type 2 diabetes and other related metabolic disorders [8]. Knock-Down of 34 candidate genes resulted in sugar-dependent lethality, including *HHEX*, *THADA*, *PPARG*, *KCNJ11*. For 23 regions, the KD of at least one candidate gene resulted in sugar-dependent toxicity [23]. Moreover, at several loci more than a single candidate gene demonstrated phenotypes when knocked down, suggesting that the SNP marked a region where several genes with similar function reside. These analyses demonstrated the utility and feasibility of using *Drosophila melanogaster* KD as an experimental model for testing functionality of orthologous human genes. Revising these methodologies to investigate adiposity, we have evaluated the functional effects of KD of cis-candidate genes based on BMI-associated SNPs robustly identified by GWAS.

For 26 of the 62 (42%) BMI loci that could be screened in the fly, we found at least one gene within the +250KB radius that showed a significant change in %BF in the fly ortholog KD compared to control (for a total of 36 significant genes). By contrast, large screens of metabolism phenotypes by RNAi screens of Drosophila typically identify 5–10% candidate genes, not correcting for multiple comparisons. For example, Ugrankar et al (2015)[24] found 61 of about 650 (9%) random RNAi transgenes resulted in significant glucose elevations (S1 Table) by student t-test, p < 0.05. More relevant to our human BMI candidate region screen, in an RNAi adiposity genome-wide screen of the fly, Pospisilik et al. (2010)[6], found ~500 functional genes for adiposity from screening 10,500 open reading frames. (~5%), also using student t-test, p < 0.05 for significance (without correcting for multiple comparisons). Thus, our findings (even after multiple comparisons corrections) are considerably enriched compared to background by screening the regions containing of BMI loci. Further, the GTEx resource (www.gtexportal.org/) confirms that all 36 of the human genes for which the corresponding fly ortholog KD resulted in a significant phenotype (as shown in Fig 3), are expressed in either the brain or adipose tissue in humans. The large number of positive findings suggest that much of the biological machinery to store and retrieve fat is conserved deep in the evolutionary tree, which makes a strong argument for the utility of this kind of high throughput functional genetic screening strategies for evolutionarily conserved genes in simpler model organisms.

Some loci regions contained more than one functional gene for adiposity. For two of the index BMI SNPs, our experiment identified three nearby genes that showed %BF KD changes in the fly. The first is rs977747, for which the closest human gene is *TAL1* (the SNP is in the 3’-utr of TAL1—Table 1, locus 1). The KD of the fly ortholog of TAL1 showed a significant decrease in %BF compared to control, as did the KD for the ortholog of the nearby *CYP4A22* gene. In contrast, the KD for the nearby *FOXD2* gene showed a significant increase in %BF. In MCF7 cells, *CYP4A22-AS1* expression has been shown to stimulate *TAL1* gene expression[25]. If this holds in either the brain or fat body of the fly (as well as in the human), it could be that the KD of *CYP4A22* (ortholog) results in reduced *TAL1* (ortholog) expression, which would result in a similar phenotype to the *TAL1* KD. By contrast, *FOXD2* KO mice have been shown to have decreased PKA expression[26]. Functional studies implicate cAMP–PKA in initiation of vascular and hematopoietic differentiation of embryonic stem cells via recruitment of the transcriptional activator cAMP response element–binding protein (CREB) to the Etv2 promoter, resulting in up-regulation of among other genes, *TALl1*[27]. This would be consistent with the idea that KD of *FOXD2* might in turn reduce PKA expression, which would in turn overexpress *TAL1*, resulting in an opposite phenotype to the KD for *TAL1* (increase rather than decrease in %BF). On the other hand, both *TAL1* and *FOXD2* are transcription factor genes, and *FOXD2* is listed as one of the many targets of *TAL1*, so the direction of causality may be from *TAL1* to F*OXD2*. Another explanation, would be that all 3 of these genes operate independently on %BF, and in the human, the proximity of the genes in the genome means that the lead SNP is tagging a common haplotype with critical regulatory variants for each. These are testable hypotheses to follow up in future experiments.

Many of the positive findings (11 of the 26 regions with at least one significant KD gene, 42%) implicate the influence of the nearest gene to the BMI lead SNP on adiposity in the fly. As our KDs were specific only to brain and adipose tissue, these results provide powerful preliminary data for further detailed experiments on the mechanism of action for these genes as the drivers of the human BMI findings.

But perhaps the most scientifically useful outcome from our screen is the identification of 10 human BMI regions where the closest gene to the BMI locus was testable in the fly and did not show a fat phenotype, but one or more other nearby genes did show a significant fat phenotype (Table 1). This “non-nearest gene” case represents 16% of the human BMI loci that could be screened in the fly. Furthermore, our screen identifies one or more strong, alternative candidates for additional functional study. Specifically, as shown in Table 1, our screen would suggest that for human BMI SNPs rs11057405, rs205262, rs9925964, rs9914578, rs2287019, rs11688816, rs13107325, rs7164727, rs17724992, and rs299412, the functional genes may NOT be the nearest ones (*CLIP1*, *C6orf106*, *KAT8*, *SMG6*, *QPCTL*, *EHBP1*, *SLC39A8*, *ADPGK /ADPGK-AS1*, *PGPEP1*, *KCTD15*, *respectively*), as these were all negative in our Drosophila screen. Instead, our screen suggests that different nearby cis gene may be more fruitful for further functional follow up for obesity, namely *ZCCHC8*, *VPS33A*, *RSRC2; SPDEF*, *NUDT3; SETD1*, *VKORC1; SGSM2*, *SRR; VASP*, *SIX5; OTX1; BANK1; ARIH1; ELL; CHST8*, respectively. Furthermore, there are an additional 5 BMI SNP regions, for which the nearest gene could not be tested in the fly (no ortholog or no available KO RNAi Fly line), but one or more nearby gene(s) did show a significant KD phenotype in the fly. These findings would suggest that for these 5 BMI SNPs rs4787491, rs7138803, rs6465468, rs16907751, and rs758747, it might be worth investigating the nearby genes *YPEL3/PAGR1*, *RACGAP1*, *PDK4*, *ZNF704* and *CLUAP1* (respectively) instead of (only) the nearest genes INO80E, *BCDIN3D/BCDIN3-AS1*, *ASB4*, *ZBTB10* and *NLRC3* (respectively).

For two of the 10 cases where a distal gene was significant rather than the nearest gene, and one of the 5 for which the nearest gene could not be tested in the fly, bioinformatic databases confirm that the SNP is an eQTL for the significant distal gene in humans. BMI SNP rs9914578 is intronic to gene *SMG6*, but that gene’s fly ortholog did not show a significant fly phenotype. However, that SNP is an eQTL for the distal gene *SRR* in adipose tissue (P = 8.1e-7) according to GTEX, and this is one of our 36 significant genes. Similarly, BMI SNP rs4787491 is intronic to gene *INO80E* (which had no available KO RNAi Fly Line), but the SNP is an eQTL for *YPEL3* in both adipose tissue (P = 3.0e-26) as well as in the human brain (P = 1.6e-7) according to GTEX, and that gene is significant in our fly screen. Finally, BMI SNP rs11688816 is intronic to *EHBP1*, but this gene’s ortholog KD did not show a significant change in %BF (1.28+0.19 vs.1.35±0.45 for control). However, the nearby *OTX1* ortholog KD did show a significant %BF phenotype compared to control (2.70±0.17 vs. 1.35±0.45, respectively). The NESDA NTR Conditional eQTL Catalog (https://eqtl.onderzoek.io/index.php?page=info) which provides eQTL results from human whole blood expression studies, confirms that this BMI SNP is a significant cis eQTL for *OTX1* (False Discovery Rate<1.3e-5), but is not a significant eQTL for *EHBP1* itself[28]. Also, Westra *et al* (2013)[29] found that rs11688816 regulates *OTX1* with a *P* = 1.8E-24 in whole blood. This evidence in humans combined with our RNAi tissue specific KD results in the fly, strongly suggest that the nearby distal genes, not the ones in which the SNP actually resides, are the genes which are conferring obesity risk in humans.

In summary, our Drosophila screen has demonstrated evidence that a high percentage of human obesity loci may be evolutionary conserved down to the insect (33%: 26 out of 78). For more than a third of these (N = 10), we found that the nearest gene to the BMI lead SNP did not seem to be the one that was functional in the fly, but one or more nearby genes were functional in the fly. Furthermore, functions of the genes identified in the fly affecting %BF are important in relation to brain, glucose and fat metabolism, cell proliferation and growth and contributing to transcription regulation. We have therefore identified specific, novel, better motivated biological targets for further study in the study of the genetic architecture of obesity. For those genes that are conserved in the insect, our study points the way towards further experimental approaches to more clearly define the mechanisms of action for loci already demonstrated to be relevant for humans.

### Limitations

The interpretation of the effect of a candidate gene is straightforward when a human gene—single fly ortholog exist. But ~30% of human genes do not have fly orthologs and therefore cannot be evaluated. The gene ontology matches between human and Drosophila genes in many cases is difficult, especially when many human homologs exist for a single *Drosophila* gene or there are many *Drosophila* orthologs (many-to-many). For these cases, we selected the best ortholog for KD; but assigning observed functions to specific genes was more difficult. The sensitivity of the model system might be limited due to the how accurately *Drosophila* models human obesity as measured via BMI. Also, our tissue-specific KDs interrogated gene effects only in the brain and the fat body, so we would miss effects that operated through other organs or tissues. While many fundamental processes in energy regulation are likely to be conserved, there will be complexities of human physiology that are not modeled well in insects. For selected genes, future studies will require validation in mammalian models.

## Materials and methods

We show in Fig 1 the outline of our experimental design. In Step 1, we began with a list of 78 BMI-associated loci seen in preliminary analyses of our own studies which were ultimately published in two comprehensive GWAS meta-analysis publications: Speliotes *et al*. 2010[30] and Locke *et al*. 2015[31]. All 78 loci, marked by the most significantly associated “lead” SNP, had reached genome-wide significance levels of *P*<10^−8^ in meta-analyses of multiple cohorts. In Step 2, we identified all (human) genes within 250kb (in either direction) of the 78 significant BMI associated lead SNPs, according to dbSNP build 147 of assembly GRCh37/HG19. In Step 3, using NCBI’s Entrez gene for identifying human gene symbols (http://www.ncbi.nlm.nih.gov/gene/), we identified the closest *Drosophila* orthologs to each of these human genes according to DIOPT—DRSC Integrative Ortholog Prediction Tool (http://www.flyrnai.org/diopt)[32], as well as pairwise alignments generated using BLAST of NCBI (for example: http://www.ncbi.nlm.nih.gov/homologene/?term=ADCY9). In Step 4, we obtained available RNAi knockdown lines for each of the fly orthologs from Step 3, and crossed these with flies containing tissue-specific drivers to knock down the expression of the genes only in the brain and the fat body. Finally, in Step 5, we raised the flies on a control diet, and compared the amount of fat/triglyceride in the tissue-specific KD group compared to the driver-only control flies.

### *Drosophila melanogaster* model

#### Fly stocks

RNAi stocks (listed in S1 Table) were acquired from the Vienna Drosophila Resource Center, as well as genetic background controls *w*^*1118*^ (for GD lines, VDRC #60000) and *y*^*−*^*w*^*1118*^*; P[attP*, *y*^*+*^, *w*^*3’*^*]VIE-260B* (for KK lines, VDRC #60100) ^[^^24^^]^, *Cg-GAL4* (BDSC #7011). Preference was given to KK lines, in which UAS-RNAi hairpins have been targeted to a landing site in Chromosome 2. The GD lines insert the UAS-RNAi at random positions in chromosomes X, 2, or 3. For three candidate genes (ARIH1, VIL1, and ZC3H4), the only lines available were GD in which the UAS-RNAi mapped to the X-chromosome. For these three lines, only female offspring carried the UAS-RNAi hairpin, therefore females were analyzed for metabolic studies. To increase the extent of KD, we crossed *Cg-GAL4* with a *UAS-Dcr* stock to generate *UAS-Dcr2; cgGAL4* stock that was used in all crosses to transgenic RNAi lines. Crosses were allowed to lay eggs for set periods to control for larval numbers. Flies were housed in temperature and humidity regulated incubators and kept in the dark for the entire experiment.

#### Fly media

We modified a commonly used *Drosophila* semi-defined medium as previously described ^[^^10^^]^. Briefly, we replaced all added sugars in the recipe (glucose and sucrose) with 51.3 g/L sucrose to yield 0.15 M sucrose.

#### Metabolic studies

Triglycerides were measured using the Infinity Triglycerides Reagent kit (Thermo Fisher #TR22321) on whole-insect homogenates. Ten animals were homogenized in PBS + 0.1% Tween and heated for 5 minutes at 65°C to inactivate lipases. 2 ml of this homogenate was mixed with 198 ml of Thermo Infinity Triglyceride Reagent and analyzed as per the manufacturer’s instructions. Non-esterified fatty acids were extracted with chloroform and methanol (Marshall et al., 1999), and analyzed as per the manufacturers’ instructions [NEFA-HR(2), Wako Chemicals, Richmond, VA]. Per-animal mass was measured by weighing groups of 10 animals. Each value represents at least 10 independent determinations.

*Tissue specific KD*: In pilot studies, we performed KD of 18 candidate genes using tissue specific drivers (*Cg-GAL4;* expresses in fat body and brain, *R4-GAL4*; expresses in fat body and midgut) or ubiquitous drivers (*actin-GAL4* and *da-GAL4*). We validated expression of the drivers by crossing to UAS-lacZ (S1 Fig, demonstrates brain expression by Cg-GAL4). Triglyceride content was determined in wandering male or female L3 larvae and three-day old adult male or female flies. The largest effects of KD were observed in male adult flies using the *Cg-GAL4* tissue specific drivers. Therefore, whenever possible, we used this driver KD of all candidate genes in the brain and fat body, and measured the effects of gene KD adult male flies. Given the known roles of adipose tissue and hypothalamus in regulation of appetite and metabolism, it is perhaps not surprising that the Cg-GAL4 driver produced the most marked phenotypes on percent body fat for KD candidate genes. For 3 target genes (ARIH1, VIL1, and ZC3H4), RNAi gene lines for males were not available, so we tested female flies against female controls.

### Statistical analyses

The quantitative percent body fat (%BF) distributions in the adult flies were tested against the corresponding controls using an analysis of variance model as implemented in PROC GLM (SAS v 9.4.), with Dunnett’s Multiple Comparisons test, which corrects for multiple comparisons when common control sets are used for multiple experimental conditions, to provide a 5% experiment-wise error rate.

## Supporting information

S1 Table An excel spreadsheet showing all of the data for this experiment, including the initial GWAS BMI SNPs, all nearby genes, their fly orthologs, and the results of the KDs for each othorlog. (XLSX)

S2 Table An excel spreadsheet showing validation of 36 significant BMI SNP-Gene pairs in human eQTL expression, in mouse KD experiments, and in previous literature. (XLSX)

S1 Fig Confirmation of Cg-GAL4 expression in Drosophila brain, using Cg-GAL4 crossed to UAS-lacZ. Two biological replicates are shown for each of larval and adult brains.
(TIF)
