# Supplementary figures and images for: A high throughput, functional screen of human Body Mass Index GWAS loci using tissue-specific RNAi Drosophila melanogaster crosses
Source: PLoS Genet. 2018 Apr 2;14(4):e1007222. doi: 10.1371/journal.pgen.1007222 (PMC5897035; doi:10.1371/journal.pgen.1007222)

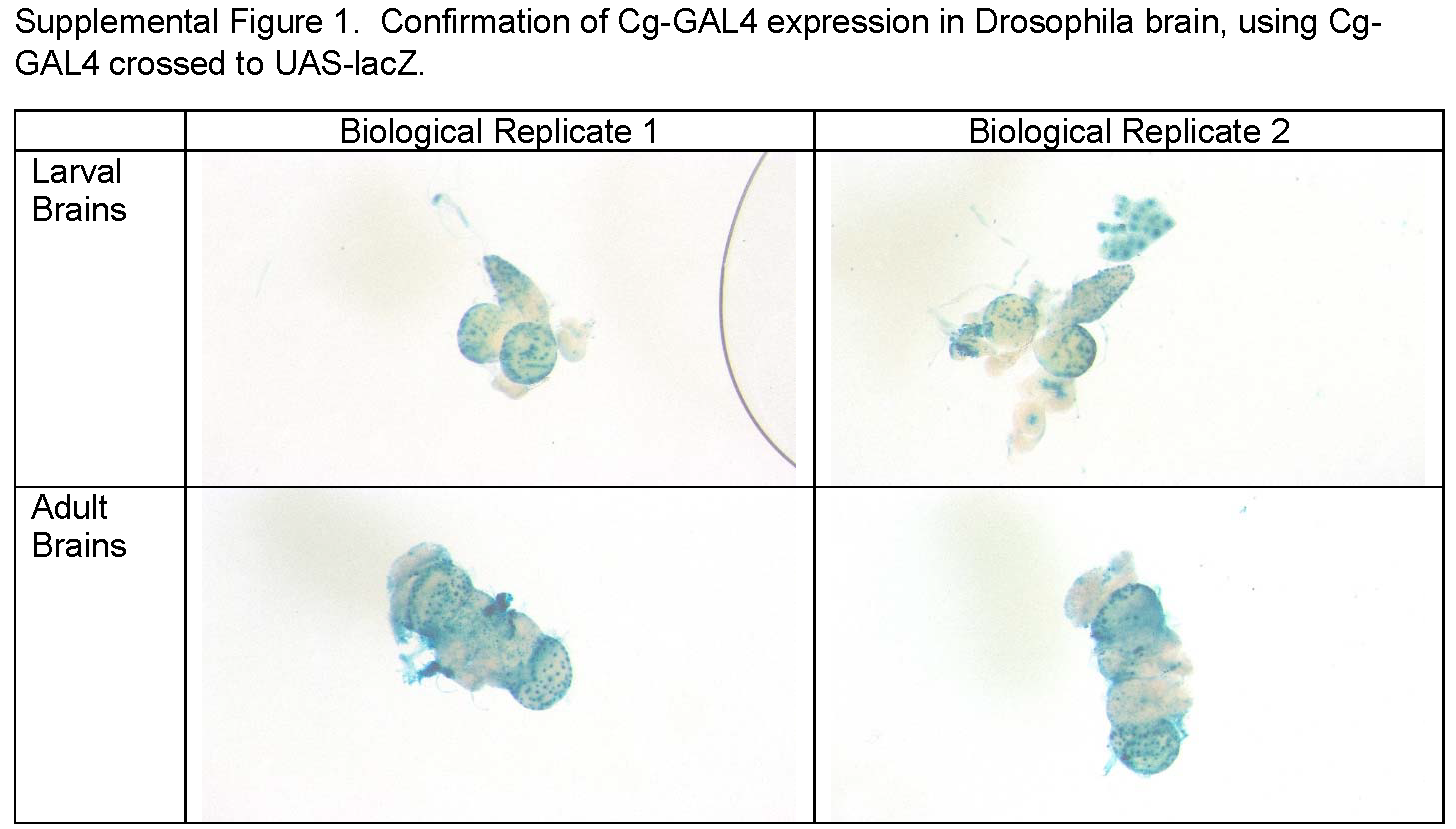

Supplement: S1 Fig — Two biological replicates are shown for each of larval and adult brains. (TIF) [file pgen.1007222.s003.tif]
